# Designer babies

**DOI:** 10.36834/cmej.68361

**Published:** 2020-03-16

**Authors:** Manish Ranpara

**Affiliations:** 1 Univerity of Toronto, Ontario, Canada

Jane and Elaine are best friends preparing a dinner in the year 2100. Jane recently gave birth to Michael, a boy with an apparently predetermined future. For a few thousand dollars, Michael’s parents purchased a genetic package named “brain and brawn,” which promised Michael Herculean strength and the intellect of Minerva*. “Good looks” were purchased as an optional extra, and insurance for genetic code failure was another dent in the wallet. “It’s an investment” the sales person explained. “The returns form a perfect child will be greater than returns earned from investments in real-estate.”

In the year 2050, the pharmaceutical industry started pumping money into the genetics field after realizing that that they could no longer sell recycled minor modifications of drugs. Realizing that people were voluntarily sending genetic material to ancestral databases and getting tested for mutations of unknown clinical significance, the industry purchased genetic information from vendors and started creating the largest ever database of genetic information. A few marketing and public relation stunts later, everyone wanted a genetically engineered baby.

“It’s called engineering because you can pick and choose sweetheart! They know the genetic profile of my entire family, and apparently I am also a descendent of Genghis Khan!” exclaimed Jane, as she explained the concept to Elaine. Like a custom-built clock, Adam and I have been able to select the genes of Michael precisely. He will be tall, handsome, and intelligent. He will be perfect. Who doesn’t want to be perfect?”

Elaine was skeptical. Growing up in a small-town community with strong religious values, she had never been the party starter. She was infamous for stirring the pot during the most sensitive moments. Her opinionated criticism did make her respected, and the debates she often started made eyebrows rise, and occasionally fists. Working part-time, she struggled to make ends meet. The thought of a market existing to design babies puzzled and worried Elaine. She viewed it as selective breeding, and an act against the natural processes of God. “What is perfect?” Elaine asked. “Doesn’t it bother you that your definition of perfect has been corrupted by the mass media? How can imperfection create perfection? Do you think it is ethical to design a baby while another baby starves elsewhere?”

Sensing a debate, Jane attempted to dodge the bullet. “I respect your point of view, but I do believe that my child will be appreciative knowing that I have done what’s best for him. Think about all the babies who could be saved from life-threatening illness at birth by genetic modification. A life is priceless.” Elaine entertained the idea for a minute, and then asked if they can also prevent non-life-threatening illnesses. “Of course!” exclaimed Jane. “They can also sell a beauty package which prevents skin moles, wrinkles and excessive cellulite from developing.”

It’s 2150 and the artificial intelligence industry has finally made the epic entrance predicted by blockbuster Hollywood movies at the turn of the millennium. People now sleep with a robotic assistant that does more than turn the lights off (the selling point is that it “satisfies every need”), and mobile phones are redundant thanks to Super PA^TM^, a pea-sized supercomputer designed as a personal assistant living in the world wide web. All jobs are performed by robots whose sole purpose is to serve their master (rumors mention a revolution). Peter is one in a family of designer baby brothers who grew-up to be 7 feet and develop a chiseled jaw. Like most people in his generation, he laughs when his grandparents call him tall, because clearly, he is average. Three of his brothers have poor health, and a lawsuit continues with the now defunct genetic industry. The industry claims that good health for designer babies was never a guarantee because of several factors including the environment. They claim it was all in the fine print. Several lawsuits later, the genetics industry invested and transformed into the robotics industry. Peter is part of this industry and he has a vision - a vision where humans are perfect. A world where humans can run faster because of robotic legs and think quicker because of robotic brains. His friends question his ideologies, but he reassures them: “People will be tall, handsome, and intelligent. They will be perfect. Who doesn’t want to be perfect?”

## Footnotes

* Terms and conditions apply. Read the small print with a magnifying glass for details, before you tick the box.

